# The Biological Structure Model Archive (BSM-Arc): an archive for in silico models and simulations

**DOI:** 10.1007/s12551-020-00632-5

**Published:** 2020-02-05

**Authors:** Gert-Jan Bekker, Takeshi Kawabata, Genji Kurisu

**Affiliations:** Institute for Protein Research, Osaka University, 3-2 Yamadaoka, Suita, Osaka 565-0871 Japan

**Keywords:** Molecular dynamics, Homology modeling, Database, Archive, Raw data, Sharing

## Abstract

We present the Biological Structure Model Archive (BSM-Arc, https://bsma.pdbj.org), which aims to collect raw data obtained via in silico methods related to structural biology, such as computationally modeled 3D structures and molecular dynamics trajectories. Since BSM-Arc does not enforce a specific data format for the raw data, depositors are free to upload their data without any prior conversion. Besides uploading raw data, BSM-Arc enables depositors to annotate their data with additional explanations and figures. Furthermore, via our WebGL-based molecular viewer Molmil, it is possible to recreate 3D scenes as shown in the corresponding scientific article in an interactive manner. To submit a new entry, depositors require an ORCID ID to login, and to finally publish the data, an accompanying peer-reviewed paper describing the work must be associated with the entry. Submitting their data enables researchers to not only have an external backup but also provide an opportunity to promote their work via an interactive platform and to provide third-party researchers access to their raw data.

The Protein Data Bank (PDB) is one of the largest collaborative scientific archives on the planet, holding the molecular structures of various biological macromolecules, such as proteins, DNA, and RNA obtained via experimental methods (Burley et al. 2019). The submitted structures were all resolved using experimental methods such as X-ray crystallography, nuclear magnetic resonance, or electron microscopy. Recently, PDB-Dev was developed as an archive to incorporate data from various experimental methods, describing structures using complementary experimental and computational techniques (Burley et al. 2017). In the past, the PDB also included several theoretical models, but they were removed more than a decade ago and later adopted by the Protein Model Portal (Arnold et al. 2009). Since then, there have been several attempts by the community at establishing an archive for computational structural biology data, in addition to more general sharing methods such as Zenodo (https://zenodo.org/). Dynameomics was developed about a decade ago and contains analysis results obtained from short MD simulations at room and high temperature for a large number of small proteins and peptides performed by the Daggett group (van der Kamp et al. 2010). Similarly, Molecular Dynamics Extended Library contains analysis results obtained from MD simulations at room temperature performed by the Orozco group (Meyer et al. 2010). Finally, GPCRmd (http://www.gpcrmd.org/) contains MD simulation results specifically for GPCR systems. Still, efforts to construct a single, public archive for raw data from computational sources have proven to be difficult.

Here, we present the Biological Structure Model Archive (BSM-Arc or BSMA) as an archive for computationally derived structural biology data. Thus, BSM-Arc for purely computationally derived data was designed to serve as the counterpart to the PDB for experimentally derived data and PDB-Dev for integrative/hybrid data. We accept a wide range of data derived via various computational methods and encourage depositors of experimental structures to the PDB that have also performed computational analysis on their structures, to also submit the data corresponding to their computational work to BSM-Arc. Depositors are free to submit their data in any format, but the data should be thoroughly documented if non-standard formats were to be used. Besides 3D structures, analysis results, either in text/binary files or in marked-up tables, can be added. Although the uploaded data files are format-free, meta-data is stored in the BSMA-STAR format, which is a format similar to the PDBx/mmCIF format, and the file can also be downloaded by the user. Meta-data such as file annotations, external database linking (e.g., to PDB and UniProt entries), and extensive descriptions can be added via an interface and are then stored in the BSMA-STAR formatted file. Thus, each BSM-Arc entry consists of a meta-data file in the BSMA-STAR format listing all the annotations, in addition to a set of raw data files uploaded by the depositor. Important to note though is that since we perform no extensive peer-review on the data and the methodology used to obtain the data, we require the data to be accompanied with a peer-reviewed paper that describes the methods used to obtain the data and a discussion of the results. Finally, for released entries, BSM-Arc incorporates viewers for 3D structures, images, and texts for standard formats, to enable users to view the data without requiring them to download the raw data.

Prospective depositors require an ORCID ID (https://orcid.org/) to submit new data. The ORCID ID enables not only the community to uniquely identify the authors of an entry but also some basic verification of the work via past achievements related to the same authors. The policies of the archive are currently very flexible and simple; the data must be related to structural biology and an accompanying peer-reviewed paper is required before publication. Although it is possible to upload data before acceptance of a paper, publication requires the data to have been discussed in a peer-reviewed paper. The data to be submitted is also free to be decided upon by the depositor. Raw data, representative data, and a combination thereof are all accepted. In case large amounts of data are submitted, it is advisable to add some additional documentation to describe the organization. For this, BSM-Arc provides several annotation methods. Multiple free-text panels can be added to an entry to add an extensive description of the data, its organization, the data formats used, a summary of the paper, etc. (Fig. 1). New entries can also be initialized from a BSMA-STAR formatted file, so that depositors can pre-set various meta-data. Files can be easily uploaded in parallel via a web interface at high speeds, so that large files can also be submitted. Files and folders can also be individually annotated by depositors if they wish to do so (Fig. 1). Depositors can also upload a graphical abstract image, which will be shown on the entry page and with the search results. Upon completing an entry, depositors can mark an entry for release, and after checking the entry for potential issues by one of our biocurators (primarily to check whether an appropriate peer-reviewed paper has been associated), the entry will be released immediately, assuming no issues were found. After release, entries can be modified by the depositors, but need to be rechecked by a biocurator upon re-release.

**Fig. 1:**
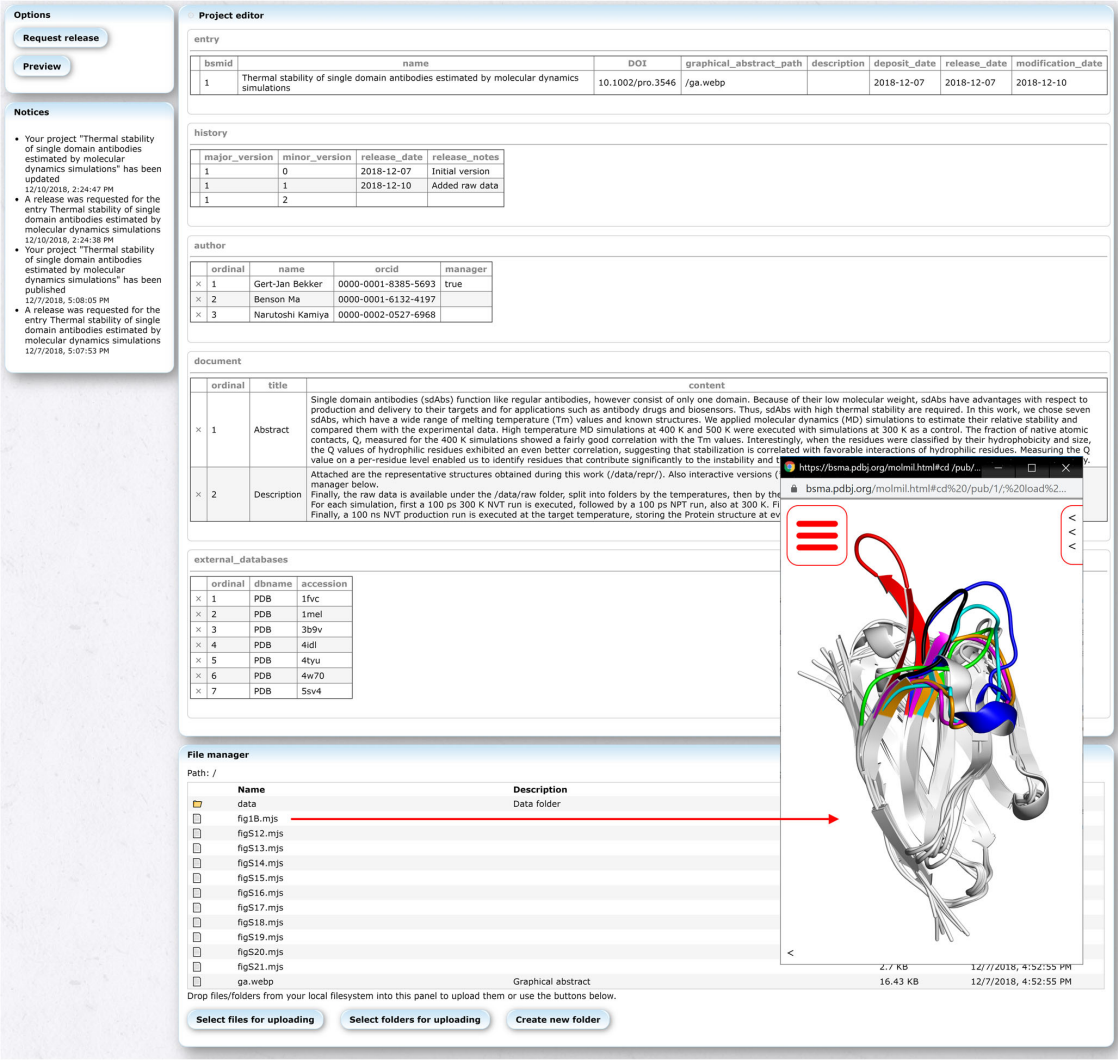
Editor/submission tool showing BSM-00001. The top-center panel (named “Project editor”) can be used to add meta-data to the entry and add extensive descriptions via full-text panels. The bottom-center panel (named “File manager”) can be used to upload new files (either via drag-and-drop operations or via the buttons) and assign per-file/folder annotations (description). Double clicking on supported files opens them in the BSM-Arc viewer (e.g., the file *fig1B.mjs* is shown in the bottom-right corner), while double clicking on folders accesses the clicked folder. Right clicking shows a context menu from which, e.g., the description can be modified and the files downloaded

Previously, Protein Data Bank Japan (PDBj) developed its own WebGL based molecular viewer, Molmil (Bekker et al. 2016), which has been integrated into many of our services (Kinjo et al. 2017, 2018). BSM-Arc also integrates Molmil for the visualization of submitted 3D structures and MD trajectories. A file manager enables users to quickly explore the submitted files, including any potential descriptions set by the depositors (Fig. 3). Double clicking on structural files will automatically open these files using Molmil. In addition, BSM-Arc also supports scripted *mjs* files, Molmil’s custom scripting format (Bekker et al. 2016), which is a mix between pymol-commands (Schrödinger 2015) and raw JavaScript code. This enables complex styling and annotation of the 3D structures and could be used to present the figures shown in the accompanying paper in an interactive manner. It also enables depositors to prepare movies, by loading a combination of structure (e.g., *gro* or *pdb* files) and trajectory (e.g., *xtc* or *trr* files) files. Molmil can also be embedded into the free-text panels, so that extensive descriptions can be combined with elaborate and interactive representations of the corresponding molecules.

Several entries have already been submitted to BSM-Arc, in various formats, sizes, and annotation styles. BSM-00001, BSM-00002, BSM-00003, BSM-00004, BSM-00006, BSM-00007, and BSM-00009 pertain to MD simulations (Bekker et al. 2017, 2019a, b; Inaba et al. 2018; Oda et al. 2018; Numoto et al. 2018; Nagarathinam et al. 2018), while BSM-00005 pertains to molecular docking (Kawabata et al. 2017) and BSM-00011 and BSM-00012 to homology models (Ishizuka et al. 2017; Kimura et al. 2017). All the projects concerning MD simulations include representative structures, but BSM-00001 also includes all the raw trajectory data including topologies and preparation files. BSM-00009 also includes trajectory files, but only of the final production run. Because of the large number of files for BSM-00001, some file/folder description is included for the higher-level folders, while in addition, a general description of the entire project is given in a free-text panel. BSM-00001, BSM-00002, BSM-00004, and BSM-00007 also contain interactive versions of the images included in the corresponding papers via Molmil script files. BSM-00005, BSM-00006, BSM-00011, and BSM-00012 make extensive use of per-file annotations to explain the nature of the data files of the entries. New entries can be submitted before releasing them in case the paper has not yet been accepted yet, e.g., to refer to the BSM-Arc entry from your paper. This has been done for BSM-00008 (Bekker et al. 2020) and BSM-00010, which were registered before completing peer-review. Then, after the paper has been published, the DOI can be assigned and the entries can be released. This is similar to the HPUB status (hold until publication) found in the PDB. Thus, a wide range of data submission and annotation styles can be used with the archive, and newer ones can be added based on feedback from the community.

Upon release, entries become immediately available and searchable (Fig. 2). In addition to the standard keyword-based search, we have also implemented a low-level SQL search methodology to enable users to easily search for specific meta-data of the released entries, similar to the PDBj Mine 2 RDB (Kinjo et al. 2017, 2018). Users can access individual entries to find more information provided by the depositors, or download the raw data files (Fig. 3). BSM-Arc entries are also cross-linked with PDB entries on the PDBj website, given that the depositors have added the corresponding annotation.

**Fig. 2:**
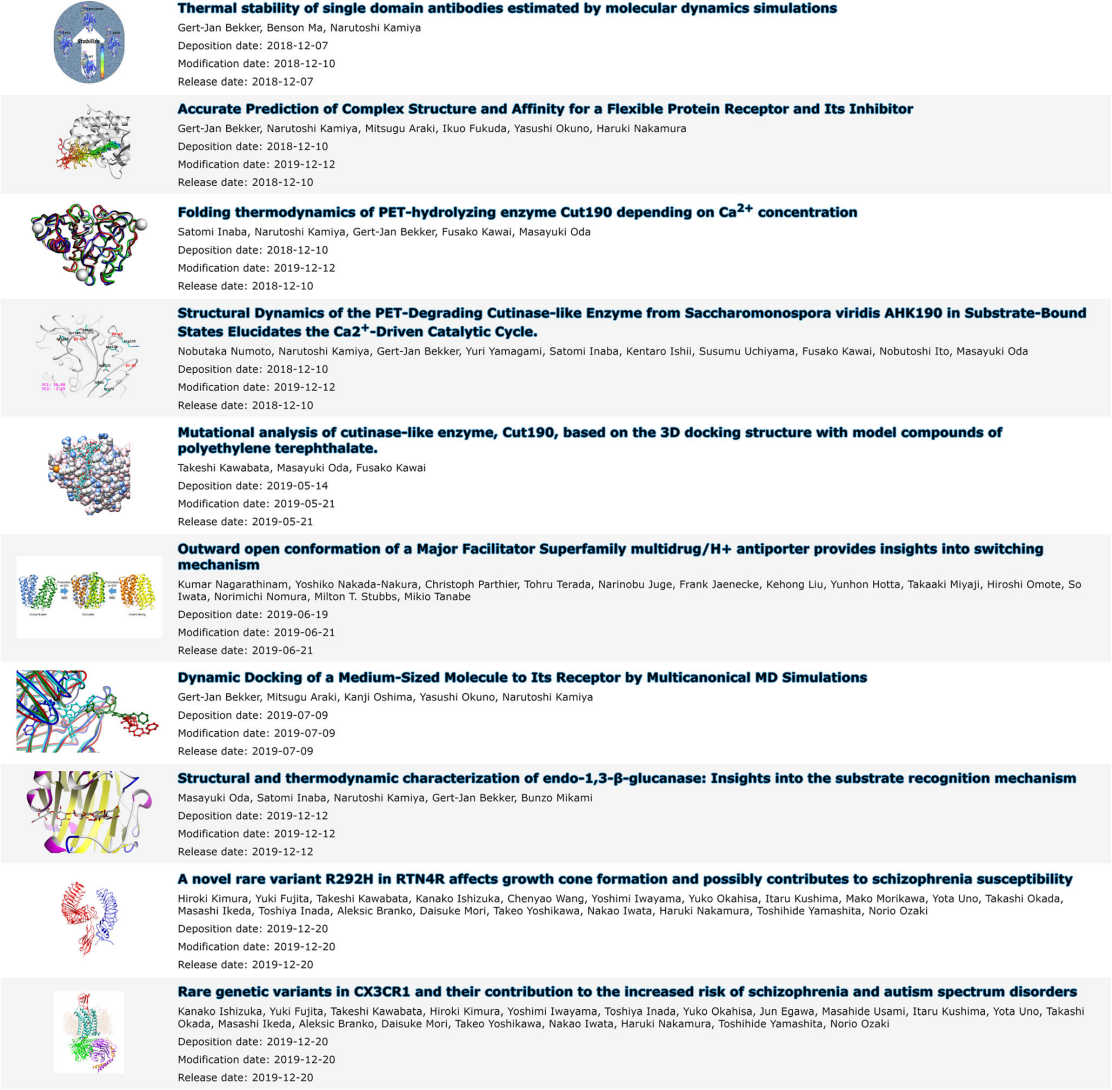
List of published entries at https://bsma.pdbj.org/search/bsma. Published entries are shown as their title, the authors, a graphical abstract set by the depositors, and the deposition, modification, and release dates

**Fig. 3:**
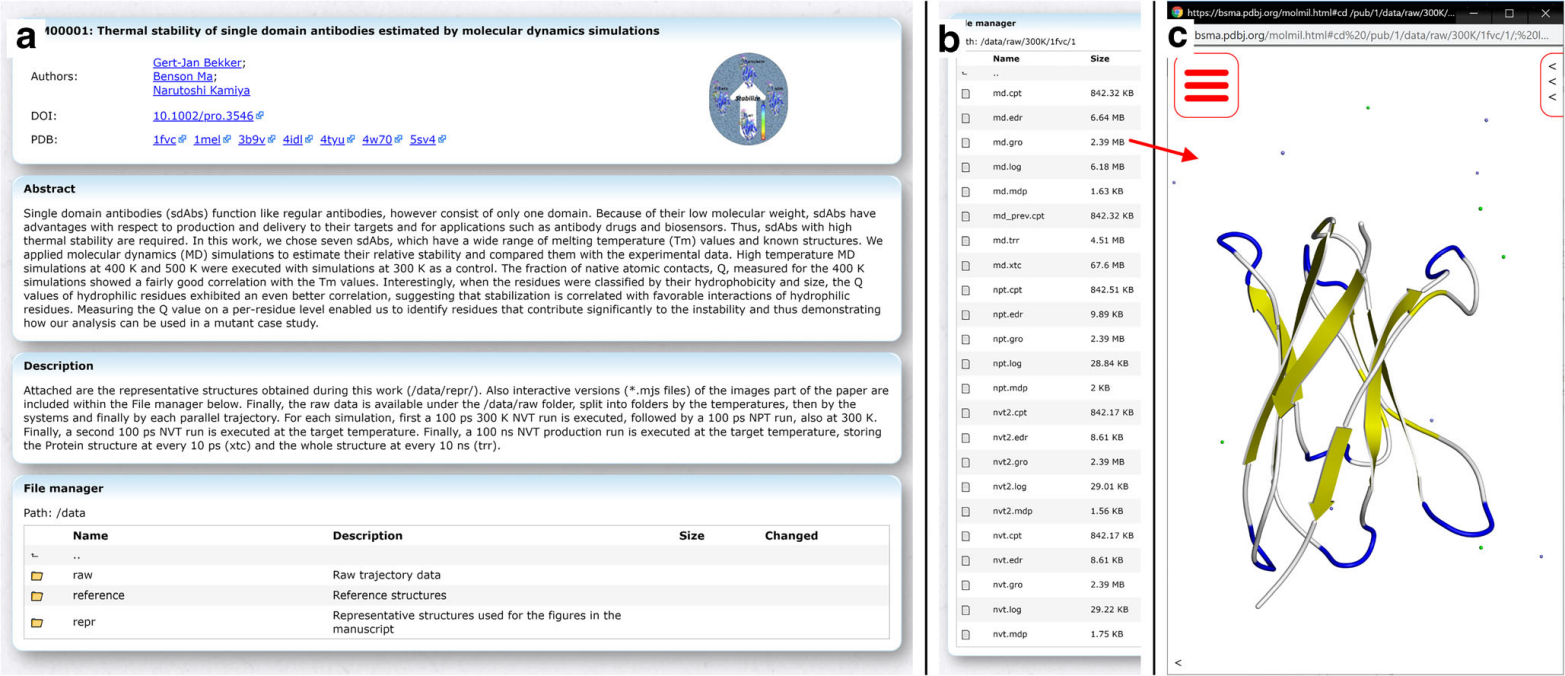
Published entry BSM-00001 at https://bsma.pdbj.org/entry/1. **a** In the top panel, the title, graphical abstract, authors, DOI, and links to external databases are listed. Below that, the free-text panels configured by the depositors are shown and finally the file manager, which works as the file manager described in Fig. 1, except no files can be uploaded and no modifications can be made. Here, two methods of annotation are used, first via a free-text panel (named “Description”), which describes the general layout of the uploaded data. Secondly, for the major files and folders, a per-file or per-folder description is included in the “File manager” panel. **b** List of raw data files included in one of the raw data folders of the entry (https://bsma.pdbj.org/entry/1/path/data/raw/300K/1fvc/1). The input and output files (both ASCII and binary) to/from the MD software were uploaded as is, without any modifications. For this entry, the individual trajectory files (*md.xtc*) were outputted during the simulation without solvent, making the trajectory files relatively small (albeit that there are 250 such trajectories in this entry). **c** The file *md.gro* loaded using the integrated Molmil viewer. In order to load a trajectory file (e.g., *md.xtc*) from this state, Molmil’s command line must be used, which can be accessed by clicking on the “<” icon in the bottom-left corner. From here, entering the command “load md.xtc” will download and load the file. Finally, to play the trajectory, the “mplay” command can be used

BSM-Arc is still only in its infancy, with many of its policies and features being quite basic. We have implemented multiple basic methods for annotation to allow depositors to freely find and use their own style. Although in the future, we would like to unify everything under a single style, first a consensus within community must be reached. We would like to invite the wider computational community to try and evaluate our archive, to help us shape it, like the experimental community has for done for the PDB.

## References

[CR1] Arnold K, Kiefer F, Kopp J (2009). The protein model portal. J Struct Funct Genom.

[CR2] Bekker G-J, Nakamura H, Kinjo AR (2016). Molmil: a molecular viewer for the PDB and beyond. J Cheminform.

[CR3] Bekker G-J, Kamiya N, Araki M (2017). Accurate prediction of complex structure and affinity for a flexible protein receptor and its inhibitor. J Chem Theory Comput.

[CR4] Bekker G-J, Araki M, Oshima K (2019). Dynamic docking of a medium-sized molecule to its receptor by multicanonical MD simulations. J Phys Chem B.

[CR5] Bekker G-J, Ma B, Kamiya N (2019). Thermal stability of single-domain antibodies estimated by molecular dynamics simulations. Protein Sci.

[CR6] Bekker G-J, Fukuda I, Higo J, Kamiya N (2020) Mutual population-shift driven antibody-peptide binding elucidated by molecular dynamics simulations. Sci Rep 10:1406.10.1038/s41598-020-58320-z 10.1038/s41598-020-58320-z PMC6989527 31996730

[CR7] Burley SK, Kurisu G, Markley JL (2017). PDB-Dev: a prototype system for depositing integrative/hybrid structural models. Structure.

[CR8] Burley SK, Berman HM, Bhikadiya C (2019). Protein Data Bank: the single global archive for 3D macromolecular structure data. Nucleic Acids Res.

[CR9] Inaba Satomi, Kamiya Narutoshi, Bekker Gert-Jan, Kawai Fusako, Oda Masayuki (2018). Folding thermodynamics of PET-hydrolyzing enzyme Cut190 depending on Ca2+ concentration. Journal of Thermal Analysis and Calorimetry.

[CR10] Ishizuka K, Fujita Y, Kawabata T, Kimura H, Iwayama Y, Inada T, Okahisa Y, Egawa J, Usami M, Kushima I, Uno Y, Okada T, Ikeda M, Aleksic B, Mori D, Someya To, Yoshikawa T, Iwata N, Nakamura H, Yamashita T, Ozaki N (2017). Rare genetic variants in CX3CR1 and their contribution to the increased risk of schizophrenia and autism spectrum disorders. Translational Psychiatry.

[CR11] Kawabata T, Oda M, Kawai F (2017). Mutational analysis of cutinase-like enzyme, Cut190, based on the 3D docking structure with model compounds of polyethylene terephthalate. J Biosci Bioeng.

[CR12] Kimura H, Fujita Y, Kawabata T, Ishizuka K, Wang C, Iwayama Y, Okahisa Y, Kushima I, Morikawa M, Uno Y, Okada T, Ikeda M, Inada T, Branko A, Mori D, Yoshikawa T, Iwata N, Nakamura H, Yamashita T, Ozaki N (2017). A novel rare variant R292H in RTN4R affects growth cone formation and possibly contributes to schizophrenia susceptibility. Translational Psychiatry.

[CR13] Kinjo AR, Bekker G-J, Suzuki H (2017). Protein Data Bank Japan (PDBj): updated user interfaces, resource description framework, analysis tools for large structures. Nucleic Acids Res.

[CR14] Kinjo AR, Bekker G-J, Wako H (2018). New tools and functions in data-out activities at Protein Data Bank Japan (PDBj). Protein Sci.

[CR15] Meyer T, D’Abramo M, Hospital A (2010). MoDEL (molecular dynamics extended library): a database of atomistic molecular dynamics trajectories. Structure.

[CR16] Nagarathinam K, Nakada-Nakura Y, Parthier C (2018). Outward open conformation of a major facilitator superfamily multidrug/H+ antiporter provides insights into switching mechanism. Nat Commun.

[CR17] Numoto N, Kamiya N, Bekker G-J (2018). Structural dynamics of the PET-degrading cutinase-like enzyme from *Saccharomonospora viridis* AHK190 in substrate-bound states elucidates the Ca ^2+^-driven catalytic cycle. Biochemistry.

[CR18] Oda M, Inaba S, Kamiya N (2018). Structural and thermodynamic characterization of endo-1,3-β-glucanase: insights into the substrate recognition mechanism. Biochim Biophys Acta - Proteins Proteomics.

[CR19] Schrödinger LLC (2015) The PyMOL Molecular Graphics System, Version 1.8

[CR20] van der Kamp MW, Schaeffer RD, Jonsson AL (2010). Dynameomics: a comprehensive database of protein dynamics. Structure.

